# Perioperative blood pressure and heart rate alterations after carotid body tumor excision: a retrospective study of 108 cases

**DOI:** 10.1186/s12871-022-01917-w

**Published:** 2022-12-03

**Authors:** Si Chen, Jingjing Xu, Guangchao Gu, Yuelun Zhang, Jiao Zhang, Yuehong Zheng, Yuguang Huang

**Affiliations:** 1 Department of Anesthesiology, Peking Union Medical College Hospital, Chinese Academy of Medical Science & Peking Union Medical College, 100730 Beijing, China; 2 State Key Laboratory of Complex Severe and Rare Diseases, Peking Union Medical College Hospital, Chinese Academy of Medical Science and Peking Union Medical College, 100730 Beijing, China; 3 Department of Vascular Surgery, Peking Union Medical College Hospital, Chinese Academy of Medical Science & Peking Union Medical College, Dongcheng District, 100730 Beijing, China; 4 School of Medicine, Tsinghua University, 100084 Beijing, China; 5 Medical Research Center, Peking Union Medical College Hospital, Chinese Academy of Medical Science & Peking Union Medical College, 100730 Beijing, China

**Keywords:** Carotid body tumor, Blood pressure, Heart rate, Perioperative management, Complications

## Abstract

**Background:**

Arising from chemoreceptor cells, carotid body tumors (CBTs) are rare neoplasms associated with hemodynamics. Perioperative changes in blood pressure (BP) and heart rate (HR) are not completely understood.

**Methods:**

This retrospective, observational, controlled study included all CBT patients from 2013 to 2018 in Peking Union Medical College Hospital. Perioperative changes in BP/HR within or between unilateral/bilateral/control groups were investigated. Perioperative details across Shamblin types were also assessed.

**Results:**

This study included 108 patients (116 excised CBTs). The postoperative systolic BP and HR increased in both unilateral (mean difference of systolic BP = 5.9mmHg, 95% CI 3.1 ~ 8.6; mean difference of HR = 3.7 bpm, 95% CI 2.6 ~ 4.9) and bilateral (mean difference of systolic BP = 10.3mmHg, 95% CI 0.6 ~ 19.9; mean difference of HR = 8.4 bpm, 95% CI 0.5 ~ 16.2) CBT patients compared with the preoperative measures. Compared with control group, the postoperative systolic BP increased (difference in the alteration = 6.3mmHg, 95% CI 3.5 ~ 9.0) in unilateral CBT patients; both systolic BP (difference in the alteration = 9.2mmHg, 95% CI 1.1 ~ 17.3) and HR (difference in the alteration = 5.3 bpm, 95% CI 1.0 ~ 9.6) increased in bilateral CBT patients. More CBT patients required extra antihypertensive therapy after surgery than controls (OR = 2.5, 95% CI 1.14 ~ 5.5). Maximum tumor diameter, intraoperative vascular injury, continuous vasoactive agent requirement, total fluid volume, transfusion, estimated blood loss, operation duration, postoperative pathology, overall complications, and intensive care unit/hospital lengths of stay significantly varied among Shamblin types.

**Conclusion:**

CBT excision may be associated with subtle perioperative hemodynamic changes. Perioperative management of CBT patients necessitates careful assessment, full preparation and close postoperative monitoring.

**Supplementary Information:**

The online version contains supplementary material available at 10.1186/s12871-022-01917-w.

## Background

Carotid body tumors (CBTs) are very rare head and neck neoplasms consisting of chemoreceptor cells, with an estimated incidence of 1/1,000,000 to 7.5/1,000,000 [1]. It has been universally accepted that complete surgical removal is the only proven cure for CBTs. Typically thought of as a key peripheral chemoreceptor, the carotid body plays an important role in control of the cardiovascular system via chemoreflexes and baroreflexes [2]. Activation of chemoreceptive cells is a powerful stimulator of the sympathetic system and has been linked with the development and progression of cardiovascular diseases, such as hypertension [3]. Moreover, a previous study has suggested that CBTs might also have an “underestimated” neuroendocrine-mediated influence on blood pressure (BP) [4]. However, how the tumor affects patient BP and heart rate (HR) remains unclear and controversial in humans. Alterations in BP and HR after CBT excision, especially after bilateral excision, are not completely understood.

First proposed in 1970, the Shamblin classification, a three-group classification system based on operative risk, has been widely used for risk stratification before surgical interventions for CBTs [5, 6]. Shamblin type I tumors do not compromise carotid vessels, and excision can be easily performed with little difficulty. Type II tumors adhere to or partially surround vessels, and excision can be difficult. Type III tumors are large and intimately surround or encase vessels [7]. The excision of type III tumors is much riskier.

Our center have effectively treated patients from the entire northern part of China and even nationwide for years. The primary objective of this research was to investigate the perioperative alterations in BP and HR in patients who underwent CBT excision. Our hypothesis was that compared with other noncarotid surgeries, CBT excision may affect both BP and HR in the short term, which may have certain clinical impacts. The secondary objective was to summarize and assess the perioperative management details of CBT patients using the Shamblin classification.

## Methods

### Study design

This investigation was a controlled, retrospective single-center study approved by the PUMC Hospital Institutional Review Board (IRB; No. S-K1180, 29 April 2020). The requirement for written informed consent was waived by the IRB. All data related to patients and operations were collected from the Hospital Information System (HIS) of PUMC Hospital. The intraoperative information regarding the included patients was obtained from the anesthetic recording system. This manuscript adheres to the applicable *Strengthening the Reporting of Observational studies in Epidemiology* (STROBE) guidelines.

### Participants

All surgical cases involving CBTs from May 1, 2013, to April 30, 2018, were included without exclusion criteria. Control cases were randomly selected from all surgical departments during the same time period to compare perioperative BP and HR alterations between cases and controls from the general population. Patients who met the following criteria were enrolled in the control group: age of 18 ~ 80 years, treatment with noncardiac surgery under general anesthesia, and three or more BP/HR values available from both before and after surgery. Patients with severe postoperative cardiovascular complications, such as any type of arrhythmia or shock, or receiving surgery that may have effects on BP/HR, such as functional endocrine tumor excision, or carotid surgery, including endarterectomy and CBT excision, were excluded from the control group. The control cases and unilateral CBT cases were included at a 2:1 ratio.

### Perioperative procedure

**Fig. 1:**
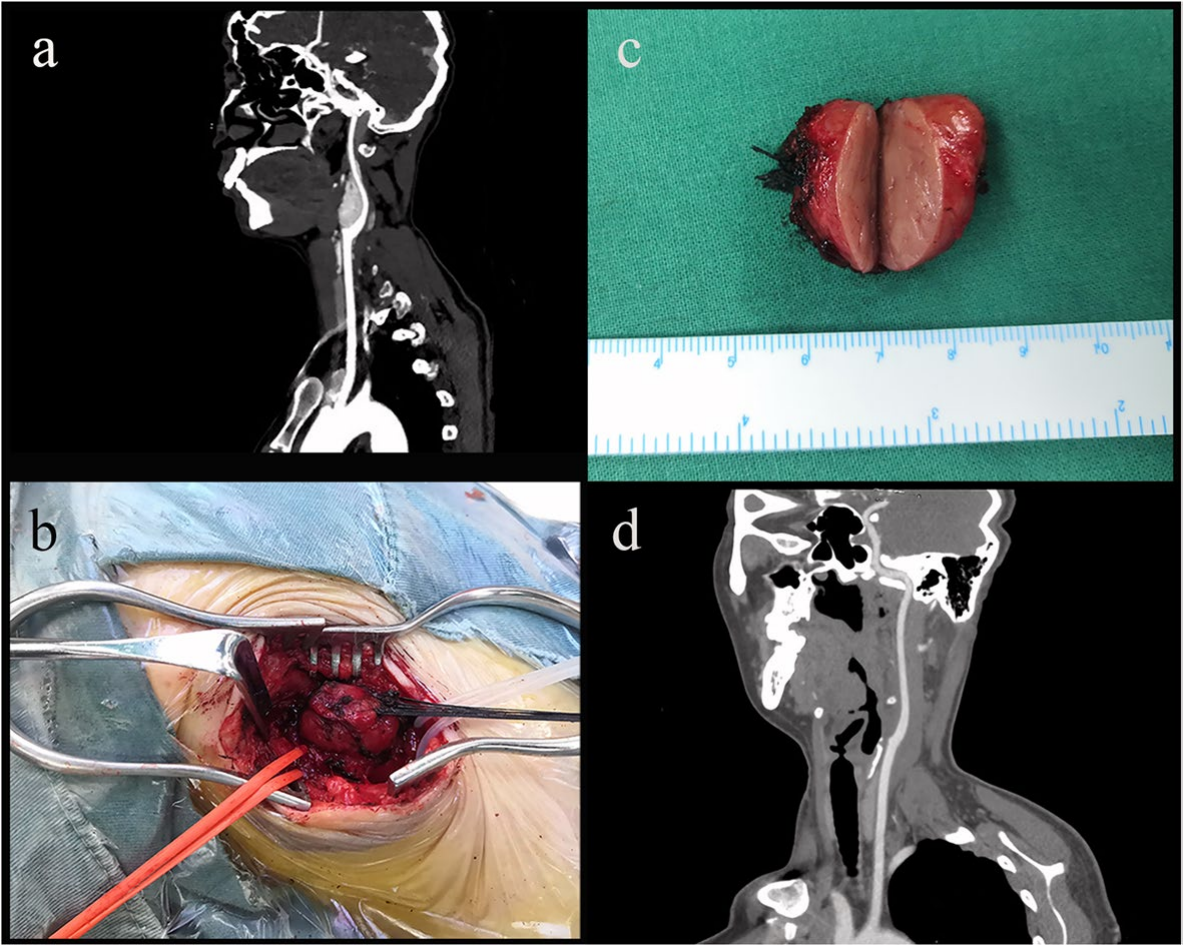
Perioperative images of a CBT patient. **a** preoperative sagittal CT scan; **b** exposed CBT with intraoperative control of the arteries; **c** demonstration of the excised CBT; **d** postoperative sagittal CT scan

**Fig. 2:**
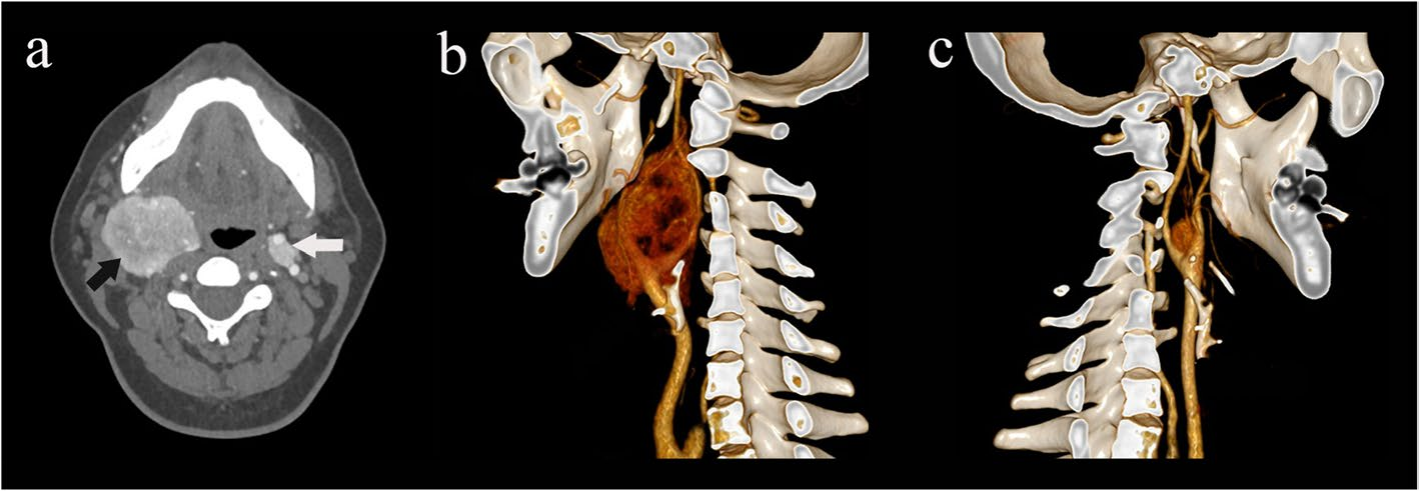
Images of a patient with bilateral CBTs. **a** preoperative transverse CT scan. Black and white arrows indicate CBTs on the right and left sides. **b**, **c** Preoperative CT angiography scan from both sides

The treatment of CBT patients at PUMC Hospital followed a general procedure (Fig. 1). Before surgery, one or two radiographic examinations, including computed tomography (CT), digital subtraction angiography (DSA, see additional file 1), magnetic resonance imaging (MRI) or Doppler ultrasound scanning, were performed for each patient for the purpose of diagnosis and classification by the Shamblin system. Preoperative catecholamine studies were conducted for patients with symptoms suggestive of inappropriate hormone secretion. Before surgery, temporary balloon occlusion of the internal carotid artery might be considered for part of patients with Shamblin type III tumor that planned for arterial ligation during surgery. All patients underwent surgery with intubation and general anesthesia. During surgery, arterial ligation, reconstruction or repair was considered according to the surgeons’ clinical experiences and technical standards. Mastoidectomy was considered for large tumors close to the skull base. For patients with bilateral tumors, smaller CBTs were operated on first. The larger ones were removed months later if no obvious contraindications developed (Fig. 2). Pre-/postoperative BP and HR data were collected every day at the same time in the morning during the hospital stay. In-hospital perioperative medication use was documented in detail. BP/HR measurements prior to hospitalization and on long-term follow-up after discharge were not available for all patients in this study.

### Definitions

In this study, each patient’s preoperative or postoperative BP/HR was the average value of the daily BP/HR measurement before or after surgery during hospitalization. For bilateral patients, the preoperative BP/HR was defined as the average BP/HR before the first operation, and the postoperative BP/HR was defined as the average value after the second operation. Extra antihypertensive agent therapy after surgery was defined as the postoperative use of additional intravenous or oral antihypertensive agents, including β-blockers, aside from the preoperative medications, or the postoperative discontinuation of preoperative antihypertensive agents during hospitalization. Family history was defined as having an immediate family member diagnosed with paraganglioma. Plateau regions were defined as any plateau province at an average elevation of more than 1000 m above sea level in China, e.g., Qinghai Province, Guizhou Province, Tibet, Inner Mongolia, Xinjiang Uygur Autonomous Region, Ningxia Hui Autonomous Region, Yunnan Province or Gansu Province. Before surgery, patient presentation of cranial nerve palsy included cranial nerve symptoms, such as dysphonia, dysphagia, hoarseness or jaw stiffness. The duration of tumor evolution was timed from tumor onset to hospital admission as reported by the patients. Surgical vascular injury was defined as either external carotid artery ligation or internal carotid artery repair/reconstruction during the surgery. Postoperative nerve dysfunction consisted of both cranial nerve and sympathetic trunk dysfunction diagnosed according to postoperative neurological symptoms including changes in voice, difficulty with tongue movement and speech articulation, difficulty swallowing and Horner’s syndrome, depending on the involved nerves. Postoperative overall complications included postoperative nerve dysfunction, wound hematoma, stroke, wound infection, and respiratory complications, among others.

### Statistical analysis

We summarized the patients’ basic characteristics using descriptive statistics. For the primary objectives of this research, changes of the pre-/postoperative BP/HR within the unilateral/bilateral/control group were conducted by paired *t* tests. Comparisons of postoperative BP/HR alterations between the unilateral/bilateral and control groups were conducted using multivariable linear regression. Considering that the different distributions of sex, age and region between the unilateral/bilateral and control groups may act as confounding factors, adjustments were made for these factors in the multivariable linear regressions. The residuals were plotted against the predicted values to check the goodness of fit of the linear models. The uniform and random distribution of points around the horizontal line at 0 was considered to indicate a suitable fit to the observations. For the secondary research objectives, to compare the perioperative details across Shamblin types, continuous variables with a skewed distribution were analyzed using the Mann-Whitney U test or Kruskal-Wallis H test. Categorical variables were compared using chi-squared tests. A two-sided *P* value less than 0.05 was considered statistically significant. Statistical analyses were conducted using SPSS 23.0 (SPSS, Inc., Chicago, IL, USA).

Because the sample size was determined by the number of patients, we calculated the statistical power following the one-factor covariance analyses used in the primary outcome assessment. Each statistical power regarding SBP, diastolic blood pressure (DBP) and HR alterations in both unilateral and bilateral patients compared with the controls was achieved (see additional file 2). Of all six powers calculated, a power > 70% was achieved for five, and > 80% was achieved for three. The statistical power of postoperative DBP alterations compared between the bilateral and control groups was 18.8%. The power of the tests was calculated using PASS 11.0 (NCSS, LLC., Kaysville, Utah, USA).

## Results

From May 1, 2013, to April 30, 2018, a total of 116 CBTs in 108 patients (34 male and 74 female) were diagnosed and excised at PUMC Hospital (Table 1). Of the 116 CBTs, all were completely excised without preoperative embolization. Temporary balloon occlusion of the internal carotid artery was carried out in seven cases (6.0%). We did not encounter any cases of functional CBTs. Of the 108 patients, mean age at presentation was 44.1 years, five patients (4.6%) had a definite family history, and 29 (26.9%) patients came from plateau regions. A palpable neck lump was the most common presentation (95 patients, 88.0%). Other symptoms included cranial nerve palsy in 13 patients (12.0%), neck pain in 12 patients (11.1%), and headache or dizziness in 12 patients (11.1%). The CBT was incidentally found during a medical examination in 5 patients (4.6%). Of the 108 patients, 100 (92.6%) had unilateral tumors, and eight patients (7.4%) had bilateral tumors and therefore underwent surgery twice. Individual information of the eight patients with bilateral tumors is shown in Table 2. After surgery, 16 cases (13.8%) required extra antihypertensive agent therapy during hospital stay, which was significantly more than the number of such patients in the control group (OR = 2.5, 95% CI 1.14 ~ 5.5, *p* = 0.024). Among these 16 cases, five patients were diagnosed with bilateral CBT, one of them received extra antihypertensive treatment after each surgery. Of the 16 cases requiring extra antihypertensive agent after surgery, 14 cases received short-term therapy and discontinued their medication before discharge. Two cases with unilateral CBT resected were discharged with the agents and continued on outpatient treatment. A total of 200 patients were enrolled in the control group. Of the 200 patients in the control group, 12 (6.0%) required extra antihypertensive therapy after surgery.

**Table 1:** Demographics and characteristics of the patients with CBT and control group

Patient demographics and characteristics	Patients with CBT (*n*=108)	Control group (*n*=200)
Age (years) (range)	44.1±10.8 (20-71)	46.8±15.8 (18-80)
Sex [n (%)]		
Male	34 (31.5)	72 (36.0)
Female	74 (68.5)	128 (64.0)
BMI (kg m^-2^)	24.0±4.1 (20-71)	23.7±3.18 (18-34)
With family history [n (%)]	5 (4.6)	/
From plateau regions [n (%)]	29 (26.9)	24 (12.0)
Preoperative hypertension [n (%)]	14 (13.0)	43 (21.5)
Tumor location [n (%)]		
Unilateral (Left)	55 (50.9)	/
Unilateral (Right)	45 (41.7)	/
Bilateral	8 (7.4)	/
Presentation [n (%)]		
Palpable neck lump	95 (88.0)	/
Cranial nerve palsy	13 (12.0)	/
Neck pain	12 (11.1)	/
Headache/dizziness	12 (11.1)	/
Incidental finding	5 (4.6)	/
Postoperative requirement of extra antihypertensive agents [n (%)]	16/116 (13.8)^a^	12 (6.0)

CBT Carotid body tumor, *BMI* Body mass index

^a^Total number is 116 because eight patients received bilateral surgeries

**Table 2:** Individual information of patients with bilateral CBT

Case ID	Age	Sex	Family history	Plateau region	Pre-SBP	Pre-DBP	Pre-HR	Post-SBP	Post-DBP	Post-HR	Extra antihypertensive agents	Postoperative complications
B1	41	M	N	N	140	89	80	133	90	81	Adalat PO	Nerve dysfunction
B2	36	F	N	N	102	67	77	117	73	93	Metoprolol PO^a^	/
B3	34	F	N	N	99	56	65	125	55	78	/	Wound hematoma
B4	42	F	N	Y	109	65	62	122	69	80	/	/
B5	36	F	Y	N	113	73	78	125	60	80	Metoprolol PO	Nerve dysfunction
B6	34	F	N	Y	116	74	75	109	63	90	Metoprolol PO	Nerve dysfunction
B7	44	M	N	N	93	58	76	105	64	87	Metoprolol PO	/
B8	43	F	N	N	111	64	89	129	80	80	/	/

*M* Male, *F* Female, *N* No, *Y* Yes, *Pre-SBP* Preoperative systolic blood pressure, *Pre-DBP* Preoperative diastolic blood pressure, *Pre-HR* Preoperative heart rate, *Post-SBP* Postoperative systolic blood pressure, *Post-DBP* Postoperative diastolic blood pressure, *Post-HR* Postoperative heart rate, *PO* Per Os

^a^Used after both surgeries

The primary outcomes are demonstrated in Table 3. For the preoperative and postoperative comparisons within groups, the postoperative SBP and HR significantly increased within the unilateral/bilateral group; however, the postoperative SBP and DBP significantly decreased within the control group. Compared with controls, the postoperative SBP significantly increased (difference in the postoperative alteration = 6.3mmHg, 95% CI 3.5 ~ 9.0, *p* < 0.001) in unilateral CBT patients after adjusting for sex, age and region, while the postoperative DBP (difference in the postoperative alteration = 1.6mmHg, 95% CI -0.7 ~ 3.9, *p* = 0.181) and HR (difference in the postoperative alteration = 0.7 bpm, 95% CI -0.8 ~ 2.1, *p* = 0.363) did not. Compared with the same control group, both SBP (difference in the postoperative alteration = 9.2mmHg, 95% CI 1.1 ~ 17.3, *p* = 0.027) and HR (difference in the postoperative alteration = 5.3 bpm, 95% CI 1.0 ~ 9.6, *p* = 0.016) increased significantly in bilateral CBT patients after adjusting for sex, age and region, while DBP (difference in the postoperative alteration = 0.9mmHg, 95% CI -5.5 ~ 7.3, *p* = 0.786) did not. The visual inspection of residual plot did not find any evidence for violating the linear regression assumptions.

**Table 3:** Perioperative alterations in baseline BP and HR in CBT patients within the unilateral/bilateral/control group

	Preoperative	Postoperative	Mean difference (95% CI)
**Patients with a unilateral tumor (** ***n*** **=100)**			
SBP (mmHg)	115.2±12.7	121.1±11.3	5.9 (3.1 to 8.6)
DBP (mmHg)	69.8±8.8	70.7±10.4	0.9 (-1.4 to 3.1)
HR (bpm)	75.6±3.8	79.4±5.5	3.7 (2.6 to 4.9)
**Patients with bilateral tumors (** ***n*** **=8)**			
SBP (mmHg)	110.4±13.3	120.8±9.1	10.3 (0.6 to 19.9)
DBP (mmHg)	68.2±9.9	69.3±10.7	1.0 (-6.9 to 8.9)
HR (bpm)	75.3±8.0	83.5±5.1	8.4 (0.5 to 16.2)
**Patients in the control group (** ***n*** **=200)**			
SBP (mmHg)	120.7±14.4	117.9±13.5	-2.8 (-4.7 to -1.0)
DBP (mmHg)	73.7±9.6	71.0±10.0	-2.7 (-4.1 to -1.3)
HR (bpm)	79.2±8.6	79.2±5.9	0.0 (-1.3 to 1.3)
**Differences between unilateral, bilateral and control groups**			
Differences in SBP	Unilateral group vs. control group		6.3 (3.5 to 9.0)
	Bilateral group vs. control group		9.2 (1.1 to 17.3)
Differences in DBP	Unilateral group vs. control group		1.6 (-0.7 to 3.9)
	Bilateral group vs. control group		0.9 (-5.5 to 7.3)
Differences in HR	Unilateral group vs. control group		0.7 (-0.8 to 2.1)
	Bilateral group vs. control group		5.3 (1.0 to 9.6)

*BP* Blood pressure, *HR* Heart rate, *CBT* Carotid body tumor, *SBP* Systolic blood pressure, *DBP* Diastolic blood pressure, *CI* Confidence interval

For the secondary outcomes, perioperative details regarding the CBT patients by Shamblin type are presented in Table 4. Maximum tumor diameter, intraoperative surgical vascular injury, intraoperative continuous vasoactive agent requirement, intraoperative total fluid volume/transfusion, estimated blood loss, operative duration, postoperative pathology, postoperative overall complications, postoperative intensive care unit (ICU) length of stay and total length of hospital stay showed significant differences between at least two Shamblin types. All the significant findings implied more severe conditions as the Shamblin type increased.

**Table 4:** Perioperative details across CBT patients based on Shamblin type (*n*=116)

	Shamblin type I (*n*=44)	Shamblin type II (*n*=27)	Shamblin type III (*n*=45)	*P* value
Preoperative assessment				
Duration of tumor evolution (months)	9.0 (4.0, 36.0)	12.0 (3.0, 60.0)	18.0 (3.5, 54.0)	0.494
Maximum tumor diameter (cm)	3.6±1.4	4.6±1.5	5.4±2.4	<0.001
Maximum tumor diameter (cm) (IQR)	3.3 (2.0, 5.0)	4.0 (4.0, 5.0)	5.0 (4.0, 6.0)	
Intraoperative management				
Surgical vascular injury [n (%)]	0 (0.0)	3 (11.1)	23 (51.1)	<0.001
Continuous vasoactive agent requirement [n (%)]	5 (11.4)	7 (25.9)	25 (55.6)	<0.001
Fluid therapy				
Total crystalloid volume (ml) (IQR)	1500 (1100, 2100)	1500 (1000, 2100)	2000 (1500, 3250)	0.029
Total colloidal volume (ml) (IQR)	0 (0, 500)	500 (0, 500)	500 (500, 1500)	<0.001
Transfusion				
RBC (ml) (IQR)	0 (0, 0)	0 (0, 0)	0 (0, 400)	<0.001
RBC (ml)	0.0±0.0	14.8±75.5	356.7±761.0	0.001
FFP (ml) (IQR)	0 (0, 0)	0 (0, 0)	0 (0, 0)	
FFP (ml)	0.0±0.0	0.0±0.0	111.0±262.0	
Estimated blood loss (ml) (IQR)	55 (0, 200)	100 (0, 300)	250 (0, 950)	0.002
Operation duration (min) (IQR)	125.0 (81.5, 138.5)	119.0 (105.0, 143.0)	207.0 (138.5, 319.5)	<0.001
Postoperative details				
Malignant pathology [n (%)]	0 (0.0)	0 (0.0)	5 (11.1)	0.016
Extra antihypertensive agent requirement [n (%)]	8 (18.2)	2 (7.4)	6 (13.3)	0.439
Complications during hospital stay [n (%)]				
Overall	17 (38.6)	10 (37.0)	28 (62.2)	0.039
Nerve dysfunction	14 (31.8)	7 (25.9)	22 (48.9)	0.098
Wound hematoma	0 (0)	2 (7.4)	1 (2.2)	0.159
Stroke	0 (0)	0 (0)	2 (4.4)	0.201
Postoperative ICU days (IQR)	0.0 (0.0, 0.0)	0.0 (0.0, 0.0)	0.0 (0.0, 18.8)	0.002
Total length of hospital stay (IQR)	14.0 (12.0, 20.0)	17.0 (12.0, 20.0)	19.0 (14.0, 24.0)	0.046

*CBT* Carotid body tumor, *IQR* Interquartile range, *ICU* Intensive care unit

## Discussion

CBTs can be classified into three distinct forms: familial, hyperplastic and sporadic. Familial types have been shown to be associated with germline mutations in three of the four succinate dehydrogenase subunit genes [8]. Unfortunately, in this study, genetic information of most of our patients was not available. Hyperplastic types are common in patients with chronic continuous hypoxia disease and patients living in plateau regions [9]. The development of tumors in the carotid body may be stimulated in these cases [1]. Sex is another risk factor for CBTs. Many other studies [1, 9, 10] have shown that females have a higher incidence of CBTs than males. Some articles have suggested that hormonal changes caused by menstruation and pregnancy and monthly blood loss through menstruation in women might be possible reasons for this difference [9]. Another hypothesis is that a larger pulmonary capacity and greater enthusiasm for sports and athletic conditioning in men may allow males to escape chronic hypoxia and account for this wide gap between the sexes [11].

Regarding the primary outcome of this study, we discovered that the postoperative BP and HR increased to varying degrees in CBT patients compared with control patients. To the best of our knowledge, this is the first study to focus on perioperative alterations in BP and HR levels compared with preoperative measures in patients who underwent CBT excision.

Peripheral chemoreceptors, including the carotid and aortic bodies, mediate the immediate circulatory and ventilatory response to hypoxemia, and their function in adults is predominantly attributable to the carotid body, which lies in close proximity to the carotid sinus baroreceptors [12]. The carotid body plays an important role in hemodynamic homeostasis by acting directly through the chemoreflex or indirectly affecting the baroreflex [3]. Originating from carotid sinus and aortic mechanoreceptors, the baroreflex buffers abrupt transient changes in blood pressure. The baroreflex can be affected by surgical damage directly to the baroreceptor or to the afferent nerve branches of the baroreflex. Iatrogenic injuries to the afferent limb of the baroreflex may result in hypertension and tachycardia [13]. The surgical excision of CBTs removes the stimulatory effect of the chemoreflex on the sympathetic nervous system; however, it may also produce concomitant baroreflex damage, counteracting the lowering effect that denervation of the chemoreflex might have on BP and HR. Although overt baroreflex failure, characterized as labile hypertension, headache, diaphoresis and emotional instability [14], occurs only in a minority of patients, baroreflex sensitivity may decrease in a large proportion of patients treated with CBT excision [15]. This may offer part of an explanation for the postoperative SBP and HR increases in this study. In recent years, there have also been studies reporting that other carotid interventions, whether endovascular or surgical interventions, especially carotid endarterectomy, correlate with an impairment of baroreceptor functions and therefore influence postinterventional BP behavior in the early postoperative phase [16–18]. These results provide evidence for our explanation of the hypothesis from another point of view. On the other hand, it has been previously reported that in conscious humans, bolus administration of stimuli given in close proximity to a carotid body leads to a decrease in HR, which is different from systemic activation of peripheral chemoreceptors, most probably as a result of the elimination of the concurrent stimulation of aortic bodies [19]. It is worth noting that besides baroreflex, the central interaction with aortic bodies may also be involved in the hemodynamic changes after the CBT resections. In this study, 16 out of 116 (13.8%) cases required extra antihypertensive agent therapy after surgery compared with the preoperative medication use, which was significantly more than the number of such patients in the control group. These results indicate the clinical impact of these changes in BP and HR. To summarize, our results suggest that postoperative baroreflex function may be affected in CBT patients. Thus, close monitoring, prompt attention and necessary treatment are essential for CBT patients’ safety.

In addition, our results revealed that compared with controls, postoperative HR alterations in bilateral CBT patients increased more by 5.3 bpm (95% CI 1.0 ~ 9.6, *p* = 0.016) after adjusting for sex, age and region, while such differences were not observed in unilateral CBT patients. We suppose it may be possible that bilateral CBT excision lead to bilateral damage to the baroreflex and chemoreflex, resulting in attenuated baroreflex sensitivity and increased hemodynamic variability, therefore causing greater HR fluctuations [20]. Previous research has reported a significant decrease in cardiac sympathetic activity in conscious rats with bilateral surgical or electrical ablation of the carotid sinus nerve [21]. Additionally, it was also demonstrated that the hypotensive response after electrical stimulation of the carotid sinus was enhanced by carotid chemoreceptor deactivation, suggesting that an intact bilateral chemoreflex counteracts the hypotensive effect of carotid sinus stimulation [22]. Therefore, close postoperative BP and HR monitoring and attention are especially recommended for patients with bilateral lesions. In this study, no functional CBTs were encountered. To some extent, secreted hormones, such as histamine, serotonin or catecholamine [23], were prevented from acting as confounding factors.

Data on the impact of the duration of baroreflex damage are limited and controversial. Previous studies have suggested that after bilateral carotid sinus denervation, BP levels markedly increased but normalized within 14 days in animal experiments; however, BP levels showed a long-term increase in all four patients treated with bilateral CBT excision [24]. In a retrospective analysis of 20 patients with hypertension, it was reported that unilateral CBT excision was associated with sustained reductions in BP 30 days after surgery [25]. In addition, alterations in the sensitivity of the baroreflex and chemoreflexes may also affect the variability in BP and HR. For instance, a previous study revealed that patients treated with bilateral CBT resection had a blunted BP response to hypoglycemia [26]. However, the identification of compensatory effects over the long term and how patients react under conditions of stress need further investigation.

Our secondary findings demonstrated that maximum tumor diameter, intraoperative surgical vascular injury, intraoperative continuous vasoactive agent requirement, total crystalloid/colloidal volume, red blood cell/fresh frozen plasma (RBC/FFP) transfusion volume, blood loss, operative duration, postoperative malignant pathology, ICU/hospital stay, and postoperative overall complications were related to Shamblin type. Some of these results were in accordance with previous studies in the literature [27–29]. According to the results, advanced Shamblin types necessitated comprehensive preparation. For instance, if required, fluid replacement for resuscitation, adequate blood products and ICU beds should be readily available. Central venous catheterization can be judiciously prepared for intraoperative continuous vasoactive agent infusion before surgery. Preoperative embolization may be considered, although embolization is currently controversial, as some studies have reported that it made no difference in reducing intraoperative blood loss [30]. Intraoperative autologous blood reinfusion can also be prepared for the reduction of allogeneic products, and leukofiltration can be conditionally considered based on the malignant potential of CBTs [31].

Regarding the intraoperative management of patients with CBT, preservation of optimal BP levels, maintenance of cerebral perfusion and optimal operating conditions for the surgeon have always been basic components [32]. For postoperative complications, associations were not observed between the Shamblin type and specific complications, such as nerve dysfunction, wound hematoma and stroke. This is in line with some studies suggesting that the Shamblin classification has limitations in predicting the occurrence of postoperative complications[33, 34].

There are several limitations to this study. First, because of the relative rarity of this disease entity, the determined sample size caused some of the analyses to be partly underpowered, especially the results related to DBP and bilateral CBT patients, and therefore increased the likelihood of false-negative results. However, as CBTs do not occur at a high frequency, the inclusion of 108 patients with 116 tumors resulted in a relatively large cohort. In addition, five out of six of the primary statistical conclusions achieved > 70% power. Therefore, we believe this study has sufficient statistical power regarding our main conclusions. Second, as this was a retrospective study, there might be unbalanced potential confounders between the groups, for instance, the complicated perioperative medication interactions, resulting in confounding effects. Third, this study was regarded as an exploratory analysis; therefore, we did not adjust the probability of type I error due to multiple comparisons in the statistical analysis. Fourth, the perioperative alterations in SBP or HR are statistically significant but numerically modest. Relationship between the hemodynamic alteration and the increased utilization of antihypertensive agents after surgery requires prospective studies with larger sample sizes and fewer untreated confounders. Fifth, we are unable to retrospectively obtain systematic, complete data from all CBT patients regarding perioperative oxygen saturation changes. Carotid bodies are the peripheral chemoreceptors that are solely responsible for the ventilator response to hypoxia [35]. Literature has reported that patients with bilateral carotid body resected may carry a risk of significant oxygen desaturation even during mild hypoxia [36, 37]. This could be of concern for patients, especially those who received bilateral surgeries and from plateau regions. Finally, all perioperative data were collected during hospitalization, which normally did not exceed three weeks. The long-term compensatory effects on BP and HR after surgery need further observation and summarization. Patients’ recovery from complications after discharge was also not investigated.

Here, we attempted to identify perioperative BP and HR alterations after CBT excision and their clinical impacts in cases compared with controls. Thus, careful assessments, full preparation, gentle operation, close monitoring and continued awareness are essential for the perioperative management of CBT patients.

## Supplementary Information

**Additional file 1. **

**Additional file 2.** 

## Data Availability

We all agree to share all the data about this article. The data will be available on Gitee (https://gitee.com/chensi93/cbtc.git).

## Abbreviations

CBT: Carotid body tumor
BP: Blood pressure
SBP: Systolic blood pressure
DBP: Diastolic blood pressure
HR: Heart rate
PUMC: Peking Union Medical College
IRB: Institutional Review Board
HIS: Hospital Information System
STROBE: Strengthening the Reporting of Observational Studies in Epidemiology
CT: Computed tomography
DSA: Digital subtraction angiography
MRI: Magnetic resonance imaging
IQR: Interquartile range
ICU: Intensive care unit
