# Supplementary figures and images for: Perioperative blood pressure and heart rate alterations after carotid body tumor excision: a retrospective study of 108 cases
Source: BMC Anesthesiol. 2022 Dec 3;22:374. doi: 10.1186/s12871-022-01917-w (PMC9719143; doi:10.1186/s12871-022-01917-w)

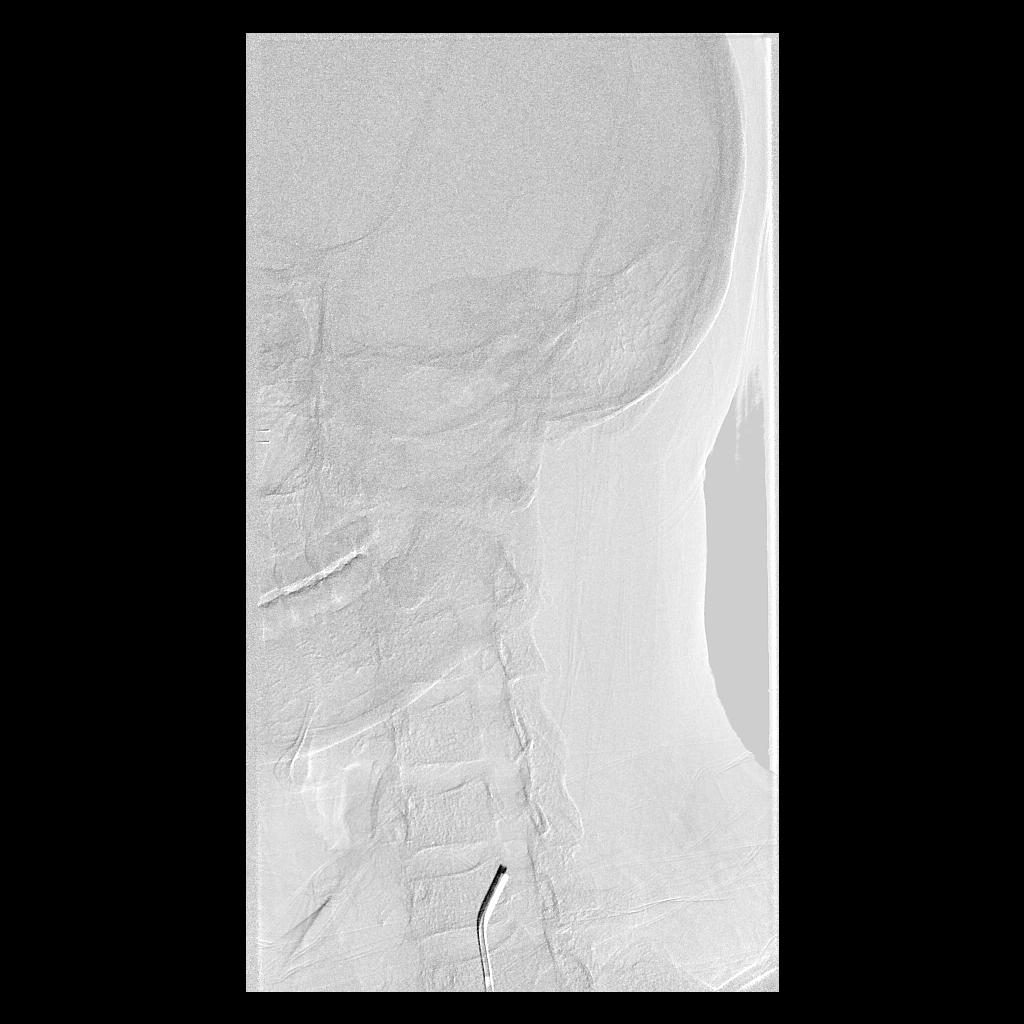

Supplement: Supplementary file 1 — Additional file 1. [file 12871_2022_1917_MOESM1_ESM.gif]
