# Supplementary material for: Perioperative blood pressure and heart rate alterations after carotid body tumor excision: a retrospective study of 108 cases
Source: BMC Anesthesiol. 2022 Dec 3;22:374. doi: 10.1186/s12871-022-01917-w (PMC9719143; doi:10.1186/s12871-022-01917-w)
Supplement: Supplementary file 2 — Additional file 2. [file 12871_2022_1917_MOESM2_ESM.docx]

Supplemental appendix 1. Statistical power regarding BP and HR alterations in both unilateral and bilateral patients compared with controls.

|  | Statistical power | |
| --- | --- | --- |
|  | Unilateral | Bilateral |
| SBP | 0.999 | 0.846 |
| DBP | 0.775 | 0.188 |
| HR | 0.967 | 0.721 |
